# Estimated indirect costs of haemodialysis versus peritoneal dialysis from a patients’ perspective at an Academic Hospital in Pretoria, South Africa

**DOI:** 10.1186/s12913-023-10109-2

**Published:** 2023-10-19

**Authors:** Kotulo Moalosi, Mncengeli Sibanda, Amanj Kurdi, Brian Godman, Moliehi Matlala

**Affiliations:** 1 Department of Public Health Pharmacy and Management, School of Pharmacy, Sefako Makgatho Health Sciences University, Molotlegi Street, Garankuwa, Pretoria, 0208 South Africa; 2 Strathclyde Institute of Pharmacy and Biomedical Sciences, University of Strathclyde, Glasgow, G4 0RE UK; 3 Department of Pharmacology, College of Pharmacy, Hawler Medical University, Erbil, 44001 Iraq; 4 Department of Clinical Pharmacy, College of Pharmacy, Al-Kitab University, Kirkuk, Iraq; 5 Centre of Medical and Bio-Allied Health Sciences Research, Ajman University, Ajman, United Arab Emirates

**Keywords:** Haemodialysis, Indirect cost, Productivity loss, South Africa, Peritoneal dialysis, Kidney failure

## Abstract

In South Africa (SA), patients with kidney failure can be on either haemodialysis (HD), which is performed by a healthcare professional in a hospital thrice weekly; or peritoneal dialysis (PD), which can performed daily at home. There needs to be more studies within the South African healthcare sector on the cost of kidney failure and especially the indirect costs associated with patients being on dialysis to provide future guidance. This study aimed to determine and compare the indirect costs associated with HD and PD from the patients’ perspective at an Academic Hospital in Pretoria. The study used a cross-sectional prospective quantitative study design. The researcher used face-to-face interviews to collect data and the human capital approach to calculate productivity losses. The study population included all patients over 18 receiving HD or PD for over three months; 54 patients participated (28 on HD and 26 on PD). The study lasted seven months, from September 2020 to March 2021. Haemodialysis patients incurred greater productivity losses per annum ($8127.55) compared to PD (R$3365.34); the difference was statistically significant with a *P*-value of *p* < 0.001. More HD (96.4%) patients were unemployed than (76.9%) PD patients.

**Supplementary Information:**

The online version contains supplementary material available at 10.1186/s12913-023-10109-2.

## Introduction

Chronic Kidney Disease (CKD) is an increasing public health burden [1–3], with associated high economic costs [4–8]. In 2017, the prevalence of CKD was estimated to be 9.1% worldwide with the highest burden of CKD is in sub-Saharan Africa, Oceania, and Latin America [9]. In 2018, kidney failure was the eighth leading cause of death among South Africans between the ages of 15 and 44, while it was the tenth cause of death among those who were aged 65 years or older [10]. There is an urgent need within countries to understand the current impact of CKD in terms of morbidity, mortality, and costs as a prelude to developing effective strategies to better manage the current situation as well as instigate future preventative strategies. This is particularly important in South Africa, with high prevalence rates for CKD, enhanced by increasing rates of,coronary vascular disease (CVD), with hypertension being a major risk condition for CVD, and diabetes along with high rates of the human immunodeficiency virus (HIV) [11–13].

CKD has different stages, with kidney failure being the last and most severe stage. A concern is increasing prevalence of kidney failure worldwide, especially in LMICs [14]. Peritoneal dialysis (PD), haemodialysis (HD), transplantation or conservative care are currently available treatment modalities for patients with kidney failure [14, 15]. Kidney transplant is the preferred method of kidney replacement therapy (KRT) [16]. However currently, there are less kidney tranplants carried out annually in the South African public sector compared to those in the private sector. The current annual rate of kidney transplantation for the public and private sector is low at 4.6 pmp (per million of population) [11]. Current obstacles to increased rates, certianly in the public sector, include a lack of resources and healthcare personnel as well as attitudes of the public and some health care workers towards organ donation [11].

In South Africa, 70.2% of patients on KRT are on HD, while 9.1% are on PD and 20.1% have a kidney transplant [17]. Haemodialysis is performed by a health professional three times a week to remove waste products [18]. However, patients can perform daily PD at home to remove waste products [18]. In view of this, PD has been shown to improve patient outcomes compared to haemodialysis (HD) in terms of both survival and health-related quality of life (HRQOL) [19–21] as well as being the most cost-effective option [22–25]. PD is also an accessible treatment option among sub-Saharan African countries because it can be managed in low-technology environments with unreliable electricity [26]. Tang et al. (2019) also suggest that PD would be suitable in countries with working-age dialysis patients where the hourly wage is of a higher value [27].

Having said this, some patients prefer HD over PD [25]. This could be a result of not being able to manage fully PD due to physical and cognitive barriers and therefore requiring some form of assistance when dialysing [25, 28, 29]. This may be why in South Africa, low HRQOL has been observed in patients undergoing PD in view of the psychological, physical, social and economic factors [30].

Assessing the costs of different treatment options is important in this population as the cost of treating patients with kidney failure has increased in several years due to an increasing rate of non-communicable diseases (NCDs), especially CVD and diabetes, increasing the burden on the national health budget [31]. We know that CKD is one of the complications of diabetes, which is a concern in sub-Saharan Africa with currently sub-optimal management of patients with diabetes [32–34]. This is a concern as the economic consequences of CKD are becoming a growing issue to patients, their caregivers, and entities responsible for payment [35], which needs to be addressed going forward. Alongside this, increasing recognition of the need to critically evaluate doses of medicines prescribed especially in hospital in patients with renal impairment [36]. The economic burden of CKD can be calculated including both indirect and direct costs [35]. Direct costs reflect the monies spent directly on treating a disease [28], while indirect or productivity costs reflect the time the patient loses for health care reasons [37].

The burden of indirect costs is more evident in patients with renal failure [35] since there is an increase in unemployment among these patients [38]. The economic and social development of the community is also affected as patients with CKD G5 between the ages of 30–60 years old and therefore part of the working population [39].

According to Yousefii et al. (2014), all costs, including direct, indirect, and intangible costs, should be considered during planning and policymaking within health systems [40]. This is important as the exclusion of indirect costs in research in South Africa may result in underestimating overall expenditure on dialysis [31, 41], which is important as South Africa is introducing universal healthcare [42]. Currently, there is limited knowledge regarding the costs of kidney failure in the public healthcare system in South Africa.

Consequently, this study aims to address this evidence gap by estimating the indirect costs of Kidney Replacement Therapy (KRT) from a patient’s perspective. The findings can be used with studies on direct costs to provide future direction to the South African authorities deciding on their future investment decisions. This is especially important with the introduction of universal healthcare in South Africa with considerable competing demands for available resources and a growing burden of NCDs.

## Methods

### Study design

The study was cross-sectional, collecting data through face-to-face interviews to estimate the indirect costs of HD vs PD.

### Study site and population

The study was conducted at a Tertiary Hospital in Tshwane, Pretoria, South Africa, which is situated in a township and caters to the population of this township and other neighbouring townships and suburbs in the Tshwane area. It is a public hospital catering for patients with CKD. A whole population study was undertaken excluding patients younger than 18 and those who had been on HD and PD for less than three months. At the time of data collection, 40 patients were on PD and 46 patients were on HD. All patients over 18 years who gave written consent to participate were interviewed.

### Data collection

Data were collected over seven months from September 2020 to March 2021 through interviews conducted in the Renal clinic when the HD patients come for their weekly visits and the PD patients come for their monthly visits. The data was collected using an instrument that was partly developed by the researchers of this study using the methodology of a Taiwanese study [27] as the study objectives were similar to the objectives of this study. Identical to the Taiwanese study, baseline characteristics, which include demographics, comorbidities, and the cause of kidney failure, were collected together with productivity losses due to KRT -related morbidity affecting the patient. Caregiver information regarding employment status and time taken off work was obtained where applicable. Productivity losses for HD and PD patients were calculated using a formula adopted from the World Health Organization (WHO) Global TB Programme [43]. To use this formula, patients were asked to recall information regarding how many hours they believed they had lost on average with each visit to the hospital. The time lost was split into the number of hours it took patients to travel to the hospital, the waiting period in hours, the number of hours they spent hospitalised, the number of hours they spent in the hospital when they had complications which did not require hospitalisation, the number of hours they spent undergoing dialysis and the number of hours they spent to fetch medication at the pharmacy in the hospital.

### Productivity loss cost calculation

The human capital approach was used to calculate productivity losses. We choose the human capital approach because it uses simple calculations using wages as a measure of employee output. This method also has a broad scope, which includes the cost of lost productivity due to illness, disability, early retirement and absenteeism [44]. A formula was adopted from the WHO, and the Global TB programme was used to calculate productivity losses, given the lack of documentation on this subject in LMICs versus high-income countries [43, 45]. A few changes were made to the adopted formula whereby we added $${t}_{complications}$$ = Estimated average time spent at the hospital for complications that did not require hospitalisation, and $${t}_{dialysis}$$ = Estimated average time patient spends performing dialysis at home or in the hospital), our $${t}_{visit}$$_was taken as the waiting period._ As a result, the loss of time in hours was multiplied by the minimum average hourly wage rate at R122.325 per hour [46] which was equivalent to $7.48 per hour at the time the study was undertaken. The average hourly wage rate per day obtained from statistics SA [46] was used for all patients, as some were never employed to avoid recall bias.

Based on the WHO guidance, the following formula was used to calculate productivity losses [43]: ${IN}^{Dialysis,h}using\;Human\;Capital\;Approach=\left(t_{visit}\times W\right)+\left(t_{hospitalisation}\times W\right)+\left(t_{travel}\times W\right)+\left(t_{pickupdrugs}\times W\right)$  

Where:

*tvisit* = Estimated average time spent per visit, including waiting time in hours

*tℎospitalisation* = Estimated Average Hospitalisation duration in hours

*ttravel* = Estimated Average travel time in hours

*tpick up drugs* = Estimated Average time employed to pick up drugs in hours

 W = average wage rate for all working individuals in the country, which is R122.325per hour [44, 46]

### Data entry and analysis

The productivity loss cost calculated for each patient was entered for analysis on a Microsoft® Excel spreadsheet, checked for accuracy, and cleaned before analysis. Thereafter, the statistician carried out data analysis using Statistical Package for the Social Sciences (SPSS) version 25. The indirect cost of dialysis was measured from a patient’s perspective, and its value was expressed as productivity losses in United States dollars (USD). Data for patient demographics were obtained, and the results were summarised as average productivity loss per patient; used Bootstrapping to get a more representative value for the means. A chi-square analysis for the confounding variables, including age, gender, and education level, was performed to see if they affected productivity loss. The study was conducted at a 95% confidence interval, where all *p*-values less than 0.05 were considered significant.

### Ethics considerations

Before executing the study, the protocol was reviewed by the Sefako Makgatho Health Sciences University Research Ethics Committee (SMUREC/P/19/2020:PG) for ethical clearance. A letter of intent explaining the aims and objectives of the study was sent to the Chief Executive Officer of DGMAH and the head of the Renal unit before collecting data. The participants were also given a consent form to sign before being interviewed. The confidentiality of the participants was maintained by not including their names and identity numbers on the data collection sheet.

## Results

### Patient demographics

The study included 54 patients, with the majority female (53%) (Table 1). Just about half of the patients were on HD (52%). Most patients were unemployed (96.4%). For most patients, the reason for unemployment was incapacity due to Kidney Failure. The confounding variables in Table 1 did not affect the productivity loss significantly besides the variable “Reason for unemployment” (*p* = 0.0034).

**Table 1:** Demographic characteristics of patients between September 2020 and March 2021

	**Patient’s Character**	**Haemodialysis patients (*N* = 28); n (%)**	**Peritoneal dialysis patients (*N* = 26); n (%)**	***P*-Value**
**Gender**	Male	12 (43%)	13 (50%)	0.610
	Female	16 (57%)	13 (50%)	0.610
**Age**	18–24	1 (4%)	1 (4%)	1.000
	25–34	2 (19%)	5 (7%)	0.198
	35–44	10 (35%)	9 (36%)	0.939
	45–54	11 (23%)	6 (39%)	0.207
	55–64	2 (19%)	5 (7%)	0.198
	65 +	2 (0%)	0 (7%)	0.158
	Mean (± SD)	44.71 (9.732)	43.3 (10.58)	0.612
**Education level**	None	1 (3.6%)	0 (0%)	0.333
	Primary School	6 (21.4%)	1 (3.8%)	0.056
	High School	14 (50.0%)	16 (61.5%)	0.400
	Tertiary	7 (25.0%)	9 (34.6%)	0.444
**Employment**	Unemployed	27 (96.4%)	20 (76.9%)	0.035
	Employed	0 (0%)	3 (11.5%)	0.067
	Self-employed	1 (3.6%)	3 (11.5%)	0.272
**Reason for unemployment**	Kidney Failure	23 (85.2%)	19 (95%)	0.236
	Not Kidney Failure	4 (14.8%)	1 (5%)	0.236
**Caregiver present**	Yes	18 (64.3%)	13 (50%)	0.293
	No	10 (35.7%)	13 (50%)	0.293
**Causes of ESRD**	ARVs	3 (10.7%)	3 (11.5%)	1
	Diabetes	1 (3.6%)	1 (3.8%)	1
	HBP	10 (35.7%)	18 (69.2%)	0.131
**Comorbidities**	HIV	1 (3.6%)	3 (11.5%)	0.317
	Diabetes	20 (71.4%)	21 (80.8%)	0.876
	HIV	10 (35.7%)	4 (15.4%)	0.109

### Average productivity loss comparison

Table 2 presents the productivity losses for each variable, which have been added to the overall yearly productivity loss for HD and PD patients. Table 2 also shows whether the results between HD and PD for each variable were statistically significant. Time spent during dialysis was high for both HD (4.00 h three times weekly) and PD (2.55 h daily) patients, significantly contributing to annual productivity losses. Overall, the productivity loss in USD of dialysing was significantly higher for HD patients ($4306.14) than PD patients ($1527.62) with a *P*-value of < 0.001. PD patients experienced less productivity losses due to appreciably less time spent travelling than HD patients, who had to go to the hospital thrice weekly, significantly increasing their average productivity loss. However, PD patients lost a significant amount of time per month compared to HD patients due to being admitted to the hospital (47.68 h) or having complications (1.35 h).

**Table 2:** Variables considered when calculating the average annual productivity loss from the patient’s perspective

Variables	Average productivity loss for HD patients (*N* = 28)		Average productivity loss for PD patients (*N* = 26)		The mean difference in productivity loss between HD and PD patients per year in (USD)	*P*-Value
	**Average time lost (Hours)**	**Average productivity loss per year (USD)**	**Average time lost (Hours)**	**Average productivity loss per year (USD)**		
Time lost in hours while waiting for services at the hospital	0.16	169.55	1.73	154.99	14.56	0.781
Time lost on days admitted in the hospital per month in hours	26.87	795.10	47.68	1357.26	-562.16	0.372
Time lost in hours while visiting the hospitals as a result of complications	0.68	20.09	1.35	39.94	-19.85	0.373
Time lost performing dialysis in hours	4.00	4306.14	2.55	1527.62	2778.52	*P* < 0.001
Time lost in hours travelling to the hospital for dialysis or to collect dialysis materials	2.59	2787.84	2.40	215.65	2572.19	*P* < 0.001
Time lost in hours picking up medicine	0.54	48.83	0.78	69.87	-21.04	0.090
Total time lost in hours	34.84	8127.55	56.49	3365.34	4762.21	*P* < 0.001

## Discussion

This is the first conducted in South Africa to assess the indirect costs associated with different forms of dialysis among patients with CKD. The results from this study show that patients with PD had lower productivity losses than those with HD, which is seen as beneficial along with lower direct costs, as documented in the study by Makhele et al. (2019) and generally [22, 31, 47]. Overall, the study highlighted that patients on HD incurred higher indirect costs and experienced the highest unemployment rate compared to those on PD. Secondly, while patients using PD incurred less costs than those undergoing HD, there were issues related to PD. These included increased hospitalisation days and visits due to complications. A study undertaken in Tygerberg Hospital in Cape Town also found that the patients on PD verbalised more symptoms related to kidney failure and the complications of their dialysis modality compared to patients on HD [48]. Our findings though that there were higher hospitalisation costs among PD compared with HD patients contradict those from Sweden [49]. We are not sure of the reasons for this; however, it could be due to issues such as training, proper sanitation at home and a regular supply of electricity to fully undertake PD [50]. Concerns with sanitation and a continual supply of electricity are especially in the informal settlements in urban cities, where most of our patients reside. In 2019, only 83.1% of households in Tshwane had access to improved sanitation, and 95.1% of households had access to piped or tap water [51]. Poor sanitation and lack of water could lead to infections. Patients on PD must have a toilet in the home for disposing of dialysate and cleanliness requirements generally when applying PD requires clean water [52]. A study conducted in Senegal which looked at non-infectious complications of PD reported that mechanical complications such as catheter migration and metabolic complications such as hypoalbuminemia could be corrected by having experienced staff members inserting catheters and giving good nutritional advice before serious complications arise [53].

Similar to this study, other studies have found that the indirect costs of HD are higher than those for PD, which is perhaps not surprising since PD can be performed at home daily without patients having to regularly travel to the hospital for dialysis [27, 54, 55]. Similarly, in Taiwan, patients on HD had a greater productivity loss per month compared to PD patients with greater outpatient care [27]. Patients on HD tend to be less productive because dialysis reduces the time spent on routine activities. They generally perform fewer activities than they would like because of their physical health., They may also not have the strength to fully deal with the pain during and after dialysis, negatively impacting on their productivity [56]. In addition, HD patients typically experience higher transport costs travelling to the hospital than PD patients, who typically only visit the hospital once a month and some even once every three months, especially during pandemics. The travel time to the dialysis facilities also impacts productivity, as most patients in this study had to travel two to three hours when visiting the hospital. As a result, substantially more patients on PD were employed in our study compared to those on HD, enhanced by a greater flexibility with their time for dialysis, with HD patients having to be at the hospital typically three times a week. Similarly, a study conducted in Spain found that 28% of the patients undergoing PD continued working, compared with only 13% of HD patients [54]. This is important with Muehrer et al. (2011) finding that patients on dialysis who are unemployed may well experience physical and psychological problems, including anxiety, depression, sexual dysfunction, and loss of self-esteem [38].

One of the key factors the South African government has with implementing universal healthcare is to improve access to tertiary hospital services by reducing the distance patients have to travel to access such services. However, this will take time, especially following the economic consequences of COVID-19 in South Africa [57, 58]. Increasing the number of nephrologists through increased training and distributing them evenly across the healthcare sectors, will help with earlier diagnosis and increase the option for PD [50, 59]. Their educational input should also help to reduce the fear among patients that they might not be able to manage PD at home due to physical and cognitive barriers as well as fears generally with potential home modifications [25, 28, 29]. In addition, assisting working age patients to stay employed will positively affect societal costs and their well-being [38]. Seeking to instigate good secondary prevention for patients who are in CKD stage 4 or 5 to avoid dialysis would also be beneficial [49]. This could be achieved by increasing the opportunities for early diagnosis for patients with risk factors, such as those with diabetes and hypertension, given concerns with underdiagnosis in South Africa and prescribing patients with early-stage CKD protective medicines against CVD and renal failure to avoid or slow down the progression of disease [60]. This is because NCDs are one of the key health priorities in South Africa [42, 61]. Such activities though need to be combined with measures to enhance adherence to these medicines due to ongoing concerns [62–64].

Whilst PD patients in our study had fewer caregivers, other studies have shown the need for caregivers, especially in ageing PD patients [28, 65]. Older patients will also have comorbidities such as diabetes mellitus with retinopathy and poor visual acuity, which could make it harder for them to perform PD without a caregiver [66]. Whilst our study showed that a lower number of patients had diabetes as a comorbidity or a cause for kidney failure, this could be due to the fact that there is more focus on preventing kidney failure in diabetic patients [31]. In addition, our findings were based on the replies of patients in the questionnaire rather than any formal diagnosis and may not be totally accurate This must be considered when reviewing potential policies to manage patients with CKD, especially in South Africa, where there is currently a limited number or no home visits by HCPs to assist patients with their PD, exacerbated by threats to healthcare workers [50]. It would also be beneficial to work with patients to reduce the time taken for PD if this is an issue that can be addressed with some patients spending up to four times longer to perform their PD than others. In addition, exploring ways to improve access to a continuous supply of electricity.

We are aware of a number of limitations with this study. Firstly, we only performed the study in one centre with a limited number of patients. Secondly recall bias could have affected the results of our study. Thirdly there were some disadvantages with using the Human Capital Method as11.5% of PD patients were employed; therefore, assuming the same wage for all participants this approach would underestimate the total cost in the group. In addition, the Human Capital Method also does not account for the possibility of patients being replaced at work, which is a possibility in South Africa given current unemployment rates. Another disadvantage of this method is that it measures the potential value of production loss due to sickness instead of actual loss which the friction cost method which would have accommodated this [44]. However, despite these limitations, we believe our findings are robust and do provide guidance for the future in decision making in this increasingly priority area.

## Conclusion

Patients undergoing HD incurred a higher productivity loss than those undergoing PD, although the difference was insignificant. Whilst the results for this variable were uniform for HD patients, there were substantial differences with PD patients. Some patients took as low as 1.25 h every day to perform dialysis, while others took 5 to 6 h. There are also concerns about greater hospitalisation among PD patients, which needs further exploration to reduce associated morbidity and costs. This is especially as more HD patients compared to PD patients were also unemployed due to the lack of flexibility that comes with performing HD. Overall, early detection of CKD and appropriate treatment to avoid progression to kidney failure would be the optimal cost saving approach. Such approaches, including improved lifestyles, would also be beneficial generally for patients with CVD and diabetes to prevent other complications, and we will be following this up in future research projects.

### Supplementary Information

**Additional file 1.**

**Additional file 2.**

## Data Availability

The data sets generated or analysed during this are included in this published article.
