# Supplementary material for: Estimated indirect costs of haemodialysis versus peritoneal dialysis from a patients’ perspective at an Academic Hospital in Pretoria, South Africa
Source: BMC Health Serv Res. 2023 Oct 19;23:1119. doi: 10.1186/s12913-023-10109-2 (PMC10585753; doi:10.1186/s12913-023-10109-2)
Supplement: Supplementary file 1 — Additional file 1. [file 12913_2023_10109_MOESM1_ESM.pdf]

Please use this tool to interview the participants and indicate with an (X) on the appropriate options or fill in the blank spaces accordingly.

Demographics:

1. Gender of participant:	Male	Female
2. ID number:		
3. Date of birth:		
4. Type of renal replacement therapy:	Haemodialysis	Peritoneal dialysis
5. Marital status of participant:		
6. Education level of participant:		
7. Comorbidities:		
8. Causes of End Stage Renal Disease:		

Questions to ask the patient:

1. What is your employment status?	
2. If unemployed, what is the reason for your unemployment?	
3. If employed, what is your sector of employment?	
4. How much time (hours) do you spend travelling to the hospital?	
5. How much time do you spend waiting for services at the hospital?	

6. How much time (in hours) do you spend for dialysis at the hospital (For Hemodialysis)?		
7. How much time (hours) do you spend to pick up medicines?		
8. How much time do you spend (hours) changing the dialysate at home (for peritoneal dialysis)		
9. Do you ever come to the hospital for other complications related to renal failure besides being admitted? If yes how many hours do you spend?		
10. How much time do you spend (in hours) in the hospital when admitted (the last 3 months)?		
11. Do you have a caregiver?	Yes	No
12. If yes; is your caregiver employed?	Yes	No
13. If your caregiver is unemployed, what is the reason they are unemployed?		
14. If caregiver is employed, do they have to take time off work to take care of you?	Yes	No
15. If employed how many hours does your care giver spend off duty assisting you with dialysis at home or helping you to do the dialysis at the hospital?		
16. If your caregiver is employed, what is their your sector of employment?		
